# Influence of intra‐ and interspecific variation in predator–prey body size ratios on trophic interaction strengths

**DOI:** 10.1002/ece3.6332

**Published:** 2020-06-01

**Authors:** Ross N. Cuthbert, Ryan J. Wasserman, Tatenda Dalu, Horst Kaiser, Olaf L. F. Weyl, Jaimie T. A. Dick, Arnaud Sentis, Michael W. McCoy, Mhairi E. Alexander

**Affiliations:** ^1^ GEOMAR Helmholtz‐Zentrum für Ozeanforschung Kiel Kiel Germany; ^2^ Institute for Global Food Security School of Biological Sciences Queen's University Belfast Belfast UK; ^3^ South African Institute for Aquatic Biodiversity Makhanda South Africa; ^4^ Department of Zoology and Entomology Rhodes University Makhanda South Africa; ^5^ Department of Ecology and Resource Management University of Venda Thohoyandou South Africa; ^6^ Department of Ichthyology and Fisheries Science Rhodes University Makhanda South Africa; ^7^ DSI/NRF Research Chair in Inland Fisheries and Freshwater Ecology South African Institute for Aquatic Biodiversity Makhanda South Africa; ^8^ INRAE Aix Marseille University UMR RECOVER Aix‐en‐Provence France; ^9^ Department of Biology East Carolina University Greenville SC USA; ^10^ Institute for Biomedical and Environmental Health Research School of Health and Life Sciences University of the West of Scotland Paisley UK; ^11^ Department of Botany and Zoology Centre for Invasion Biology Stellenbosch University Matieland South Africa

**Keywords:** Bluegill, context‐dependency, functional response, interaction strength, largemouth bass, piscivory, size‐scaling, tilapia

## Abstract

1. Predation is a pervasive force that structures food webs and directly influences ecosystem functioning. The relative body sizes of predators and prey may be an important determinant of interaction strengths. However, studies quantifying the combined influence of intra‐ and interspecific variation in predator–prey body size ratios are lacking.
2. We use a comparative functional response approach to examine interaction strengths between three size classes of invasive bluegill and largemouth bass toward three scaled size classes of their tilapia prey. We then quantify the influence of intra‐ and interspecific predator–prey body mass ratios on the scaling of attack rates and handling times.
3. Type II functional responses were displayed by both predators across all predator and prey size classes. Largemouth bass consumed more than bluegill at small and intermediate predator size classes, while large predators of both species were more similar. Small prey were most vulnerable overall; however, differential attack rates among prey were emergent across predator sizes. For both bluegill and largemouth bass, small predators exhibited higher attack rates toward small and intermediate prey sizes, while larger predators exhibited greater attack rates toward large prey. Conversely, handling times increased with prey size, with small bluegill exhibiting particularly low feeding rates toward medium–large prey types. Attack rates for both predators peaked unimodally at intermediate predator–prey body mass ratios, while handling times generally shortened across increasing body mass ratios.
4. We thus demonstrate effects of body size ratios on predator–prey interaction strengths between key fish species, with attack rates and handling times dependent on the relative sizes of predator–prey participants.
5. Considerations for intra‐ and interspecific body size ratio effects are critical for predicting the strengths of interactions within ecosystems and may drive differential ecological impacts among invasive species as size ratios shift.

## Graphical abstract

Body size ratios between consumers and resources are a pervasive factor in ecosystems across habitat types and taxonomic groups, and may alter the strength of trophic interactions. The present study quantifies the influence of predator–prey body size ratios systematically using functional responses, making comparisons at both the intra‐ and interspecific level. Our results suggest that consumer–resource interaction strengths are underpinned by body size, with attack rates peaking at intermediate size ratios, yet differing between predator species.

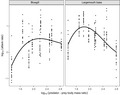

## 1 INTRODUCTION

Predation is an important factor in natural communities that exerts a significant influence on both predators and prey (Dick et al., 2017; Lima, 2002; Sih, Crowley, McPeek, Petranka, & Strohmeier, 1985). From the perspective of the predator, the ability to capture prey is critical in determining energy acquisition required for growth, reproduction, and survival (Brose et al., 2019; Hempel, Neukamm, & Thiel, 2016; Kaemingk, Graeb, & Willis, 2014; Steinhart, Stein, & Marschall, 2004). For prey, it is an important driver in the dynamics that underpin many aspects of their behavior and morphology allowing them to persist under predatory threats (Carlson & Langkilde, 2014; Preisser, Bolnick, & Benard, 2005; Werner & Peacor, 2003). As a result, a broad range of physiological, behavioral, and morphological features have been shown to affect predator–prey interactions (Lagrue, Besson, & Lecerf, 2015; Portalier, Fussmann, Loreau, & Cherif, 2019). Furthermore, predation outcomes are often dependent on the combination of certain traits of the species involved (Barrios‐O'Neill et al., 2016; Barrios‐O'Neill, Kelly, & Emmerson, 2019; Christensen, 1996; Einfalt, Parkos, & Wahl, 2015; Kolář, Boukal, & Sentis, 2019; Uma & Weiss, 2012).

Body size is an important trait that is strongly linked to foraging outcomes. For instance, in fish, bigger predators often have greater prey capture success owing to enhanced swimming abilities, improved visual acuity, and changes to morphology such as increased gape (Christensen, 1996; Graeb, Mangan, Jolley, Wahl, & Dettmers, 2006; Miller, Crowder, & Rice, 1993). Similarly, larger prey size permits greater reaction distances, faster escape speeds, and often provides size refuges from predation (Cuthbert, Callaghan, & Dick, 2019; Hansen & Wahl, 1981; Rodgers, Downing, & Morrell, 2014). However, the contributions of these variables to the interactions between predators and prey can be influenced by the relative body size ratios of the participants, which can be an important determinant in predator–prey dynamics (Asquith & Vonesh, 2012; Barrios‐O'Neill et al., 2016; Brose et al., 2019; Jennings & Warr, 2003). For example, consumers may exhibit lower attack rates toward relatively small and large resources, resulting in a hump‐shaped function that peaks at intermediate consumer–resource ratios (Brose, 2010; McCoy, Bolker, Warkentin, & Vonesh, 2011; Preston et al., 2018; but see Barrios‐O'Neill et al., 2016). As such, the relative differences in body size that are observed in interacting predator and prey species can have important implications for population structure and community stability (Otto, Rall, & Brose, 2007; Sentis, Binzer, & Boukal, 2017). Furthermore, the effects of body size ratio on trophic interactions may depend on prey densities, owing to the underlying density dependence of consumer–resource interactions (Holling, 1959).

The ability of an individual to capture resources or to avoid predation is not constant through time, but can vary as body size changes during ontogeny (e.g., McCoy et al., 2011). Many species exhibit size‐specific ontogenetic shifts in resource use, which play an important role in determining growth and survival of individuals with a subsequent influence on population‐ and community‐level processes (Reichstein, Persson, & De Roos, 2015; Rudolf & Rasmussen, 2013; Werner & Gilliam, 1984). Size‐specific changes may emerge across age classes where older, larger individuals act as separate “ecological species” (Polis, 1984; Schröder, Nilsson, Persson, Kooten, & Reichstein, 2009). However, changes may also emerge in cohorts of a similar age that exhibit large variations in size (Brooks, McCoy, & Bolker, 2013; Pfister & Stevens, 2002). Likewise, this can result in discrete stages comprised of separate size classes with a range of functional roles that are based on selection of diet (De Roos, Leonardsson, Persson, & Mittelbach, 2002; De Roos, Persson, & McCauley, 2003; Huss, Persson, & Byström, 2007; Parkos & Wahl, 2010; Rudolf & Rasmussen, 2013; Urbatzka, Beeck, Van Der Velde, & Borcherding, 2008). Most species undergo such size‐specific ontogenetic shifts in diet, often corresponding to external changes and internal factors, such as food supply or physiological demands (Werner & Gilliam, 1984). There is also much evidence to support the importance of relative body size to the outcome of these interactions between predatory fish and their prey (Fuiman, 1994; Lundvall, Svanbäck, Persson, & Byström, 1999; Scharf, Buckel, Juanes, & Conover, 1998). In many instances, prey size range can change during ontogeny, where maximum prey size increases while minimum prey size changes only slightly. This allows larger predators a greater prey size choice compared to smaller predators that are unable to feed on large prey and have thus a restricted prey size range (Scharf, Juanes, & Rountree, 2000). Energetic limitations, where a prey size does not provide sufficient energy compared with capture costs, may further influence prey selectivity as energetically costly prey may not be sustainable for the growth of predators (Portalier et al., 2019). However, there is a distinct lack of studies which examine effects of intraspecific size variations on the scaling of consumer–resource interactions.

Here, we assess the effect of variations in predator–prey body size ratios on trophic interactions and quantify the influence of both intra‐ and interspecific variations. We investigated whether changes in the relative size of two fish predators (bluegill *Lepomis macrochirus* and largemouth bass *Micropterus salmoides*) and their prey affect feeding interactions, and the relationship between prey density and predation rate (i.e., the so‐called “functional response”; Holling, 1959). Bluegill and largemouth bass are important predators in freshwaters, and both are global invaders that have been introduced extensively outside of their native range of North America. Both fishes are known to have major impacts on native communities due to predation (Almeida, Almodóvar, Nicola, Elvira, & Grossman, 2012; Ellender, Weyl, & Swartz, 2011; Godinho & Ferreira, 2000; Mittelbach, 1988). Bluegill and largemouth bass undergo ontogenic shifts in relation to resource and habitat use (Parkos & Wahl, 2010; Post, 2003; Werner & Hall, 1988), and are known to engage in piscivory (Azuma, 1992; Pelham, Pierce, & Larscheid, 2001; Taguchi, Miura, Krueger, & Sugiura, 2014). However, the window of opportunity for such changes may narrow over time because of the concurrent growth of the target fish prey (Olson, 1996).

Functional responses can be a useful means of quantifying and comparing effects of predators on prey populations under different environmental contexts (Dick et al., 2014). Both functional response form and magnitude are useful indicators of the strength of interactions between predators and their prey (Barrios‐O'Neill et al., 2016; Dick et al., 2013; Dickey et al., 2020; Sheath et al., 2018). Three functional response forms are commonly characterized (Holling, 1966): Type I forms describe a linear relationship between consumption rates and resource density, and are typically associated with filter‐feeding organisms that are not constrained by handling times (Jeschke, Kopp, & Tollrian, 2004); type II functional responses are hyperbolic, whereby most, if not all, resources are consumed at low densities and consumption rates are restricted at high densities by handling time; sigmoid type III functional responses allow for refuge at low densities as attack rate increases with prey density, before feeding rates again saturate at high densities. While the type II functional response is thought to be more destabilizing for prey populations and may lead to local extinctions, type III curves may stabilize resources (Juliano, 2001).

The aims of this study were to investigate intra‐ and interspecific resource use variations as a function of the relative predator body size of bluegill and largemouth bass toward a common fish prey species. Using functional responses, we quantified predator–prey interactions and asked whether predation rates were conserved across three size categories of both predators and prey, in a fully crossed experimental design. We predicted that larger fishes of both species would have higher maximum feeding rates of all prey sizes compared to small and intermediate predators. Similarly, we expected predators of intermediate size to be more efficient predators on smaller and intermediate prey compared to large prey, and smaller predators to be most effective at consuming smaller prey. Further, we expected functional response attack rates to peak at intermediate predator–prey body size ratios, and for handling times to lengthen under increasing ratios. In addition, given the earlier switch to piscivory in largemouth bass and the reports of their voracity as predators (Alexander, Dick, Weyl, Robinson, & Richardson, 2014), we expected impacts on prey populations to be greater in this species.

## 2 MATERIALS AND METHODS

### 2.1 Animal maintenance

We examined functional responses of two fish species, the bluegill *L. macrochirus* and the largemouth bass *M. salmoides*, toward a prey fish, the Mozambique tilapia *Oreochromis mossambicus*. Young‐of‐year bluegill and largemouth bass were collected in March 2015 by seine netting in Mosslands (33°24′7.28″S; 26°27′6.89″E) and Yarrow (33°24′55.34″S; 26°22′34.98″E) reservoirs, near Makhanda (formerly Grahamstown), South Africa. Tilapia were supplied by AquaCulture Innovations, Makhanda. All fishes were transported to the Department of Ichthyology and Fisheries Science (DIFS), Rhodes University, Makhanda, and were housed in 600‐L tanks in a closed recirculating system (water flow to the fish tanks 1 L/min; 18 ± 1°C). Fishes were allowed to acclimate to the system for at least 72 hr prior to use in predation trials.

### 2.2 Functional response trials

Experiments were conducted on three size classes of each predator that were fully crossed with three size classes of prey. Bluegill and largemouth bass were assigned to one of three size classes based on their mass (see Table 1). Trials were completed in cages with volumes scaled to length of predatory fish (approx. 1 L 4 mm^−1^; Table 1). Cages were scaled according to fish length in order to control for differences in predator–prey encounter rates that would naturally arise among size classes, therefore removing such differences as a confounding variable. Individual cages were constructed from 1.5 mm mesh and floated using buoyancy aids in 15 separate 300‐L fiberglass tanks that were part of the same flow‐through system as the holding tanks described above. This allowed five trials each of small, intermediate, and large predators to run at any one time. The order of trials was fully randomized across all treatments (predator species and size, prey size, prey density; see below). Due to the high number of trials (358 in total), fish predators were reused in the different size ratio treatments. Fish of each size were selected from a common pool of at least 60 individuals; thus, reuse occurred a maximum of two times. Fish were left for at least 2 days between trials and maintained on larval chironomids to minimize experimental prey learning. Fish predators were humanely euthanized at the end of the experiment with an overdose of clove oil.

**TABLE 1:** Mass (g ± *SE*) and length (mm ± *SE*) of largemouth bass, bluegill, and tilapia

	Size 1	Size 2	Size 3
Largemouth bass (g, mm)	1.70 ± 0.19, 50.33 ± 0.78	4.95 ± 0.18, 73.40 ± 0.51	11.38 ± 0.47, 99.0 ± 1.62
Bluegill (g, mm)	1.65 ± 0.04, 45.59 ± 0.39	5.45 ± 0.11, 70.69 ± 0.61	12.29 ± 0.46, 92.33 ± 0.71
Tilapia (g, mm)	0.018 ± 0.001, 11.05 ± 0.05	0.035 ± 0.002, 14.65 ± 0.19	0.075 ± 0.004, 18.50 ± 0.14
Cage dimensions (cm, L)	24.5 × 23.0 × 23, 12.9	27.0 × 26.8 × 26.8, 19.4	29.5 × 29.5 × 29.5, 25.6

Note

Cage dimensions (height × breadth × width cm) and volume (L), scaled for length of predator, are also presented.

Bluegill and largemouth bass of each size class (Table 1) were starved for 72 hr, before being randomly selected from their holding tanks and transferred to the appropriate size‐scaled cage (see above) 1 hr prior to a trial. Pilot observations indicated that this was sufficient time for fish to settle in cages before consuming prey. Individual fish were then presented with one of three sizes of tilapia prey (Table 1) at five prey densities (2, 4, 8, 16, 32), with at least three replicates per density. Small tilapia prey were further presented at densities of 64 prey/cage to facilitate reaching an asymptote in consumption rates. Feeding trials were run for 1 hr, after which prey consumption was enumerated based on examination of remaining live prey. Controls were three replicates (one for each cage size) of each prey size and density in the absence of predators.

### 2.3 Statistical analyses

Statistical analyses were conducted using R v3.4.4 (R Development Core Team, 2018). Differences in overall prey consumption among predator species, predator sizes, and prey sizes, and their two‐ and three‐way interactions, were assessed using a generalized linear model (GLM) with a quasi‐Poisson error distribution and log link, irrespective of prey density. This error family was selected owing to residual overdispersion. A backward stepwise deletion process was followed until all nonsignificant terms and interactions were removed, starting with the highest‐order interaction term (Crawley, 2007). Effect sizes in the resulting model were inferred using *F*‐tests via analysis of deviance (type III sums of squares). Prey densities of 64 were excluded as they were not present across all treatment groups (i.e., absent for medium and large prey). Significant effects in the model were analyzed with Tukey's contrast post hoc tests via least square means (Lenth, 2016). Statistical significance was inferred at the 95% confidence level in all analyses.

Binomial GLMs with logit links considering the proportion of prey consumed as a function of prey density were used to categorize functional responses. A significantly negative first‐order term indicates a type II functional response, while a type III functional response is categorized by a significantly positive first‐order term followed by a significantly negative second‐order term (Juliano, 2001). Where evidence for a particular functional response form was equivocal, Akaike's information criterion (AIC) was used to select models which minimized information loss, with lower values indicating a better fit (see Pritchard, Paterson, Bovy, & Barrios‐O'Neill, 2017).

As prey were not replaced as they were consumed, Rogers' random predator equation was used to model functional responses (Rogers, 1972):

(1) $$N_{e}=N_{0}\left(1-\exp\left(a\left(N_{e}h-T\right)\right)\right)$$

where *N_e_* is the number of prey eaten, *N*_0_ is the initial density of prey, *a* is the attack rate (cage/hr), *h* is the handling time (hr/prey), and *T* is the experiment duration (i.e., 1 hr). The Lambert W function was used to fit the model, owing to the recursive nature of the random predator equation (Bolker, 2008; McCoy & Bolker, 2008). Multiple estimations of *a* and *h* parameters were generated via bootstrapping (*n* = 20 per experimental group; lower cap at 0). These parameters were next compared using Gamma GLMs (structured as before).

Bootstrapped *a* and *h* parameter estimates were then analyzed using polynomial regression models separately according to fish species as a function of predator–prey body mass ratios (continuous predictor; calculated from fish group mean masses). We log_10_‐transformed *a* estimates and body mass ratios prior to analyses, and log_10_(*x* + 1)‐transformed *h* estimates as log_10_(0) is not defined and the bootstrap yielded a few null estimated values of handling time. Model comparisons were performed using AICc to select among linear, quadratic, and cubic polynomial models. Diagnostic plots were used to ensure residuals met the assumptions of parametric testing (Zuur, Ieno, & Elphick, 2010).

Owing to our nonreplacement experimental design, we tested whether prey depletion biased *a* and *h* parameter estimation from the random predator equation (Equation 1). To do so, we first simulated prey depletion during the time course of an experiment using the following population dynamic model considering a type II functional response:

(2) $$\begin{array}{c} \frac{\mathrm{dN}}{\mathrm{dt}}=-\frac{\mathrm{aNP}}{1+ahN} \end{array}$$

where *N* is the prey density,* P* is the predator density, *a* is the attack rate (cage/hr), and *h* is the handling time (hr/prey). To generate predictions of expected prey survival in the experiment, initial values of *N*_0_ and *P* were set at the initial prey and predator densities corresponding to the experimental treatments, and the population dynamic model was integrated over the time interval of the experiment using the R package “DeSolve” (Soetaert, Petzoldt, & Setzer, 2010). To mimic our experimental data, we used the population dynamic model to simulate levels of prey depletion at four experimental durations (0.2, 0.5, 1.0, 1.5 hr) across the six experimental prey densities (2, 4, 8, 16, 32, 64). Input functional response parameters at each duration were systematically varied (*a*: 0.45, 1.00, 5.00; *h*: 0.002, 0.01, 0.30), while the opposing parameter was fixed at an intermediate value (i.e., *a* = 1.00; *h* = 0.01) (*n* = 3 simulated per density). Predator densities were maintained at one. Second, the random predator equation (Equation 1) was fit to these rounded consumption simulations at each duration and parameter input separately, to estimate *a* and *h*. These estimated values were then compared to the input *a* and *h* parameters based on the overlap of their standard error for each treatment, to test whether increasing experimental duration, and thus prey depletion, caused estimation bias. We calculated prey depletion for each duration and parameter scenario by dividing the total simulated number of prey eaten by the total of initial prey densities (i.e., *N*_e_/*N*_0_).

## 3 RESULTS

**FIGURE 1:**
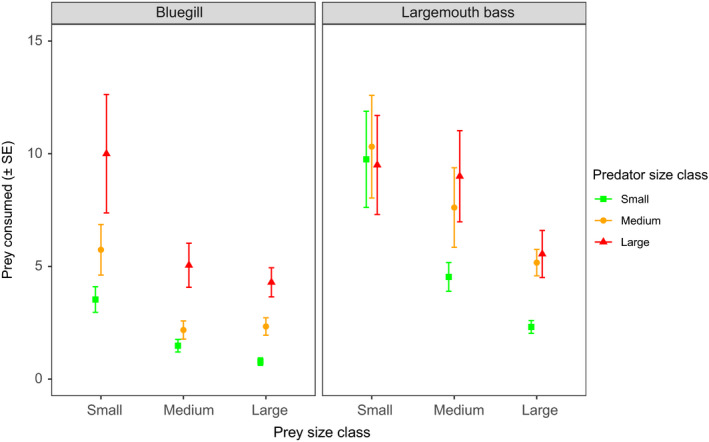
Mean (±*SE*) prey consumed of different size classes by small, medium, and large bluegill and largemouth bass pooled across ubiquitous prey densities (up to 32 ind./cage)

Control prey without predators had 100% survival in all replicates, and thus, experimental deaths were attributed to consumption by predatory fish, which was also observed. Accordingly, it was not necessary to adjust functional responses for background prey mortality (see Rosenbaum & Rall, 2018). Smallest prey were most vulnerable overall (*F*_2,331_ = 26.93, *p* < .001; Figure 1) and experienced 158% and 73% greater mean mortality than large and medium prey, respectively (Tukey's test, both *p* < .001). Medium‐sized prey were 49% more vulnerable than large prey (Tukey's test, *p* = .04). A significant interaction was found between predator species and predator size (*F*_2, 331_ = 7.28, *p* < .001; Figure 1) due to largemouth bass consuming 235% and 122% more prey than bluegill when predators were small‐ and medium‐sized, respectively (Tukey's test, both *p* < .001), yet only 27% more when both predators were large‐sized (Tukey's test, *p* = .08). There were no other statistically clear two‐way or three‐way interactions, which were thus removed during the backward stepwise deletion process.

**FIGURE 2:**
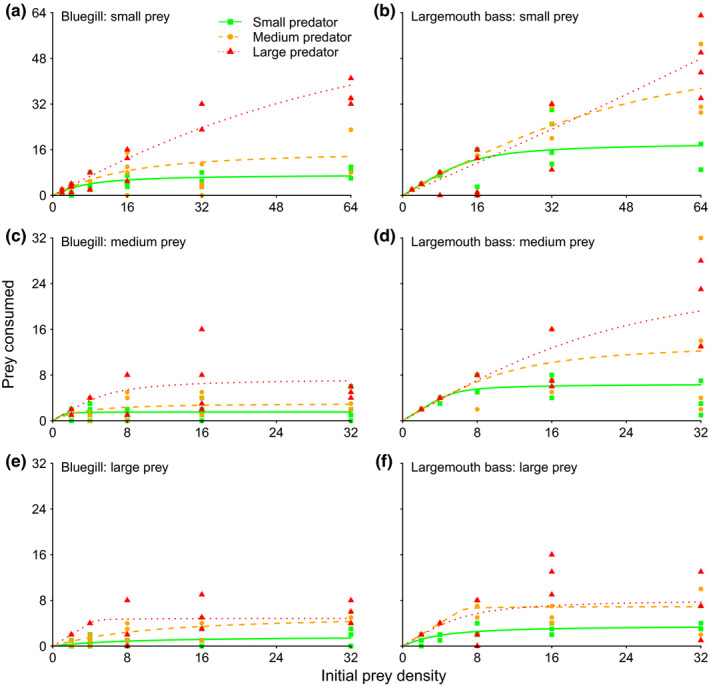
Functional responses of three sizes classes of bluegill (a, c, e) and largemouth bass (b, d, f) toward small‐ (a, b), medium‐ (c, d), and large‐sized (e, f) tilapia prey. Points are raw data. Note differences in axes scaling according to prey size

Linear coefficients considering prey consumption as a function of prey density were significantly negative, irrespective of predator species or predator and prey sizes, indicating type II functional responses (Table 2). Large largemouth bass consumption toward small prey was an exception to this owing to the lack of asymptotic prey densities (dotted curve, Figure 2b); however, the type II random predator model (Rogers, 1972) minimized information loss here as compared to the type III (Hassell 1978) and generalized models for conditions without prey replacement (both ΔAIC > 2) (see Pritchard et al., 2017). Toward small prey, largemouth bass functional responses tended to be of higher magnitude than those of bluegill when predators were small‐ and medium‐sized (Figure 2a,b). Toward medium‐sized prey, largemouth bass functional response magnitudes were generally greater irrespective of their size (Figure 2c,d). Contrastingly, functional response magnitudes were more similar in height between predators toward large prey (Figure 2e,f).

**TABLE 2:** Linear coefficients (first‐order terms) resulting from logistic regression of proportional prey consumption as a function of prey density across predator species and size classes

Species	Prey size class	Predator size class	Linear coefficient, *P*
Bluegill	Small	Small	−0.03, <.001
		Medium	−0.02, <.001
		Large	−0.02, <.001
	Medium	Small	−0.07, <.001
		Medium	−0.08, <.001
		Large	−0.11, <.001
	Large	Small	−0.05, 0.02
		Medium	−0.04, 0.01
		Large	−0.09, <.001
Largemouth bass	Small	Small	−0.06, <.001
		Medium	−0.03, <.001
		Large	0.004, 0.35
	Medium	Small	−0.15, <.001
		Medium	−0.08, <.001
		Large	−0.05, 0.003
	Large	Small	−0.07, <.001
		Medium	−0.12, <.001
		Large	−0.11, <.001

**FIGURE 3:**
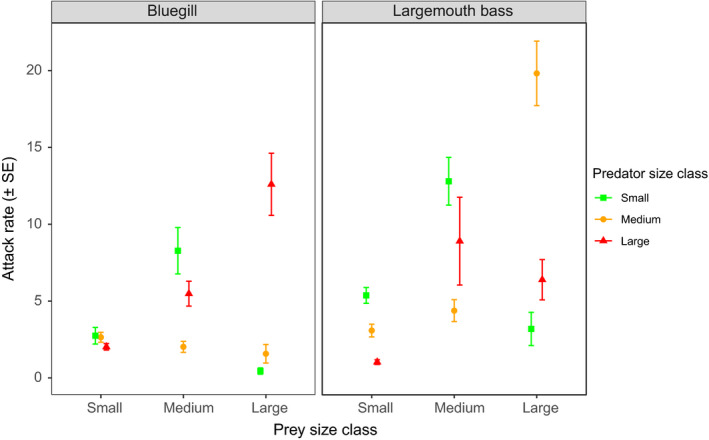
Mean (±*SE*) bootstrapped attack rates toward tilapia prey of different size classes by small, medium, and large bluegill and largemouth bass

Attack rates across sizes classes of bluegill and largemouth bass differed significantly according to prey size owing to a significant three‐way interaction between predator species, predator size, and prey size (*F*_4, 342_ = 7.42, *p* < .001) (Figure 3). Toward small prey, bluegill attack rates did not differ significantly among predator sizes (Tukey's test, all *p* > .05), while small and medium largemouth bass exhibited significantly higher attack rates than large largemouth bass (Tukey's test, both *p* < .01). Toward intermediate prey classes, small‐sized bluegill attack rates were highest, followed by large‐sized bluegill, and both were significantly greater than medium‐sized bluegill (Tukey's test, both *p* < .05). Similarly, small‐sized largemouth bass attack rates were significantly greater than that observed in medium‐sized largemouth bass (Tukey's test, *p* < .01). Toward large prey, large bluegill attack rates were significantly greater than the attack rates of either small‐ or medium‐sized bluegill (Tukey's test, both *p* < .001). In turn, medium‐sized bluegill attack rates were significantly higher than small‐sized bluegill (Tukey's test, *p* < .001). Attack rates for largemouth bass were significantly higher by medium‐sized predators compared to either small‐ or large‐sized predators (Tukey's test, both *p* < .01). Accordingly, interactions between predator and prey sizes were displayed statistically, with small‐sized predators generally exhibiting the highest attack rates toward small and intermediate prey classes, while attack rates of medium‐ and large‐sized predators were greatest toward large prey.

**FIGURE 4:**
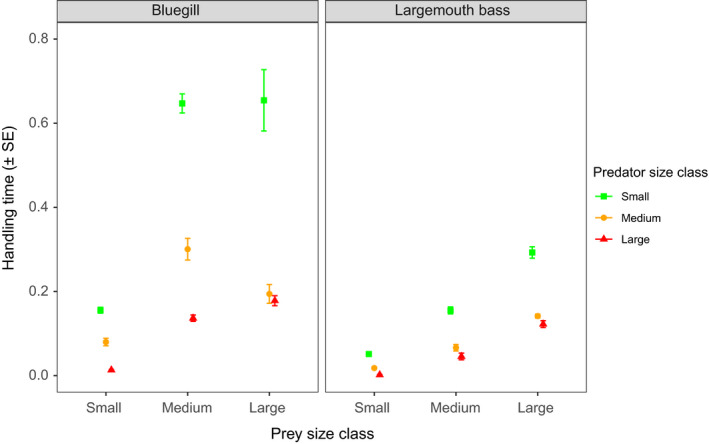
Mean (±*SE*) bootstrapped handling times toward tilapia prey of different size classes by small, medium, and large bluegill and largemouth bass

A significant three‐way interaction term also indicated differences in handling time among predator species and size classes toward differently sized prey (*F*_4, 342_ = 3.44, *p* = .01) (Figure 4). For both predator species, handling times by small predators were always significantly longer than those of medium‐ and large‐sized predators under all prey sizes (Tukey's test, all *p* < .001). Similarly, handling times of medium‐sized bluegill were significantly longer than those of large‐sized bluegill toward small‐ and intermediate‐sized prey (Tukey's test, both *p* < .001); this difference was not statistically significant toward large‐sized prey (Tukey's test, *p* = .99). Contrastingly, medium‐ and large‐sized largemouth bass handling times were only statistically different toward small prey sizes (Tukey's test, *p* < .001). Overall, while handling times generally related negatively with predator size, we found species‐specific differences, with small bluegill handling times being particularly longer than those of largemouth bass. Further, intermediate–large largemouth bass exhibited more similar prey handling capacities across prey types.

**FIGURE 5:**
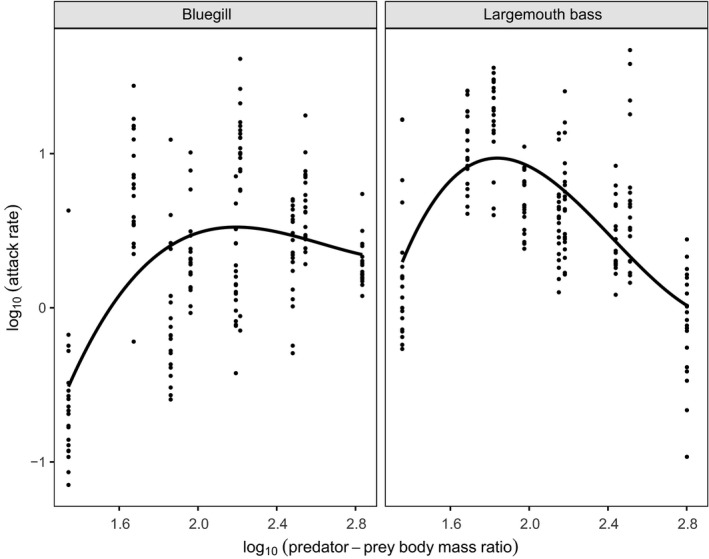
Scaling of attack rate parameter with predator–prey body mass ratios between bluegill and largemouth bass. Polynomial regression lines are presented alongside raw data points

For both predator species, scaling of attack rates followed a unimodal pattern with predator–prey body mass ratios, peaking at intermediate values (Figure 5). According to their AICc values, the best model was the cubic polynomial model for both species here (Table 3). Largemouth bass attack rates tended to reach a greater magnitude and peaked at lower predator–prey body bass ratios than bluegill.

**TABLE 3:** Linear, quadratic, and cubic coefficients resulting from polynomial regression of bootstrapped functional response parameters as a function of predator–prey body mass ratios between predator species

Species	Parameter	Term	Coefficient, *P*
Bluegill	*a*	Linear	2.77, <.001
		Quadratic	−2.98, <.001
		Cubic	0.76, 0.11
Largemouth bass		Linear	−2.14, <.001
		Quadratic	−3.53, <.001
		Cubic	1.34, <.001
Bluegill	*h*	Linear	−0.81, <.001
		Quadratic	0.15, 0.003
		Cubic	—
Largemouth bass		Linear	−0.39, <.001
		Quadratic	0.11, <.001
		Cubic	−0.06, <.001

Note

Inclusion of terms was based on ΔAkaike's Information Criterion.

**FIGURE 6:**
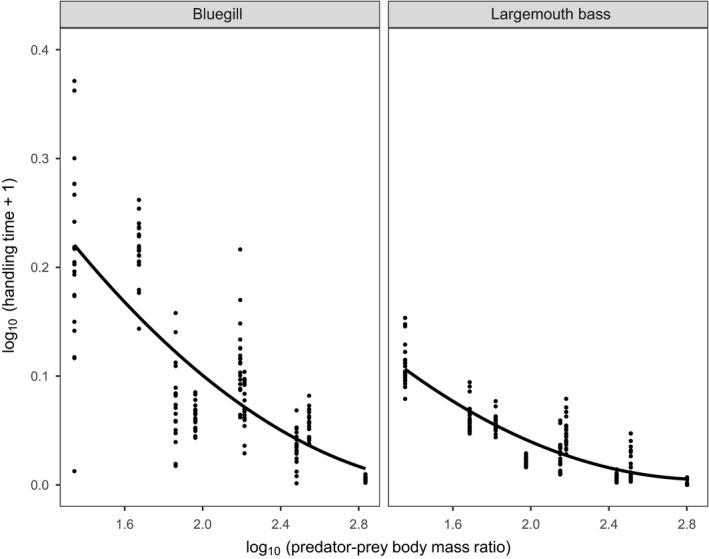
Scaling of handling time parameter with predator–prey body mass ratios between bluegill and largemouth bass. Polynomial regression lines are presented alongside raw data points

Handling times generally decreased concurrently with increasing predator–prey body bass ratios for both fish species (Figure 6). The quadratic polynomial model was the best fit for bluegill, while the cubic model was selected in the case of largemouth bass (Table 3). Largemouth bass handling times tended to be reduced across predator–prey body mass ratios compared to bluegill, and particularly under low body mass ratio values.

**Figure A1:**
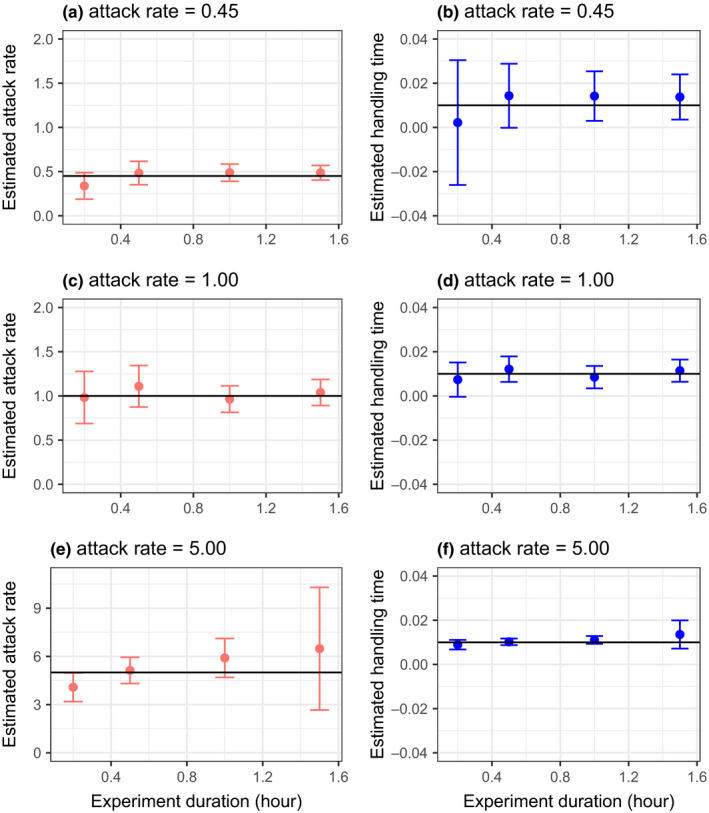
Functional response parameter estimates (attack rates: a, c, e; handling times, b, d, f) modelled from consumption simulations across different experimental durations and input attack rate values. Input handling times were fixed at 0.01. Estimates are ±*SE* and the solid lines represent the input parameter value. Note differences in axes scaling

**Figure A2:**
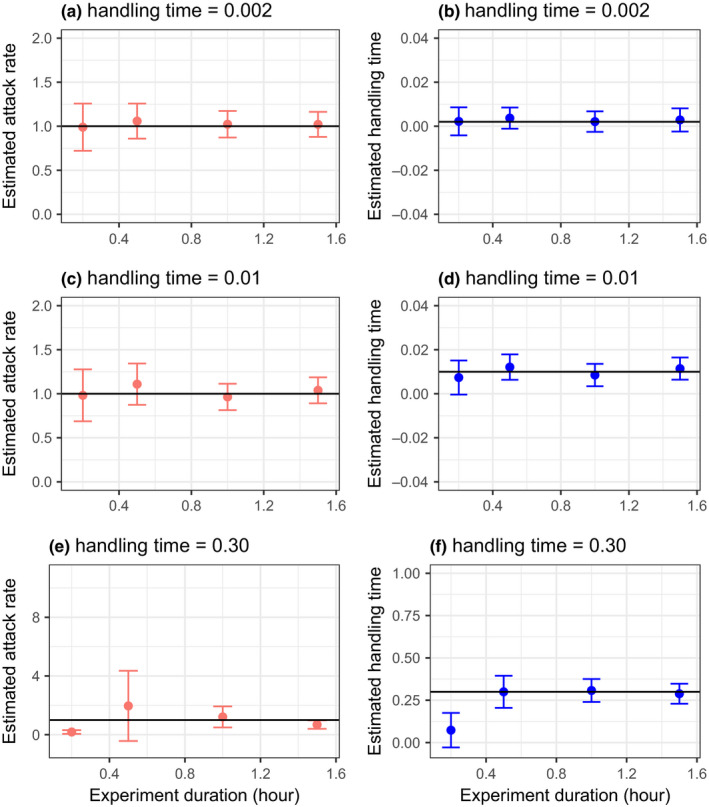
Functional response parameter estimates (attack rates: a, c, e; handling times, b, d, f) modelled from consumption simulations across different experimental durations and input handling time values. Input attack rates were fixed at 1.00. Estimates are ±*SE* and the solid lines represent the input parameter value. Note differences in axes scaling.

**Figure A3:**
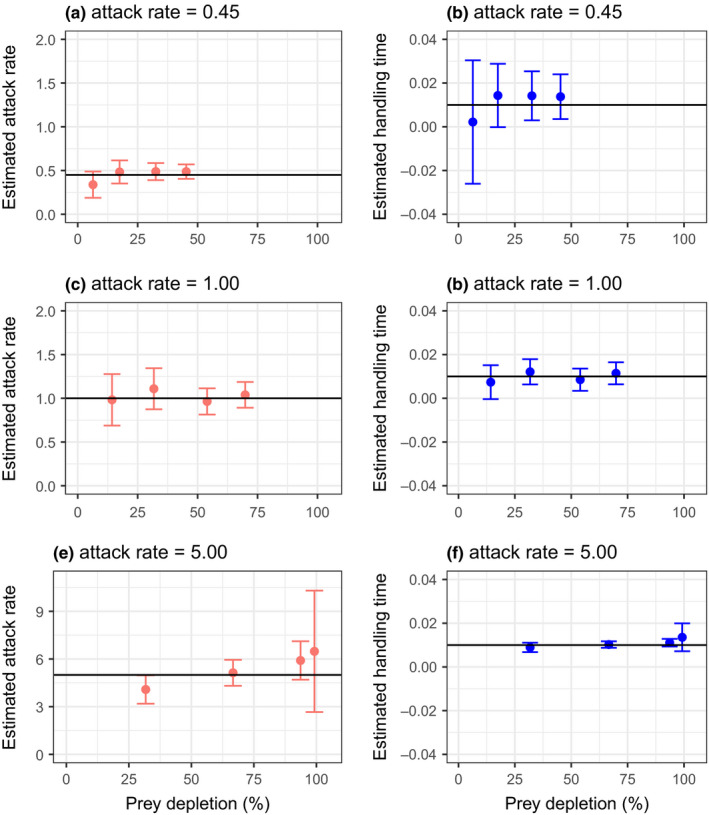
Functional response parameter estimates (attack rates: a, c, e; handling times, b, d, f) modelled from consumption simulations across different prey depletions (total prey eaten divided by total density) and input attack rate values. Input handling times were fixed at 0.01. Estimates are ±*SE* and the solid lines represent the input parameter value. Note differences in axes scaling.

**Figure A4:**
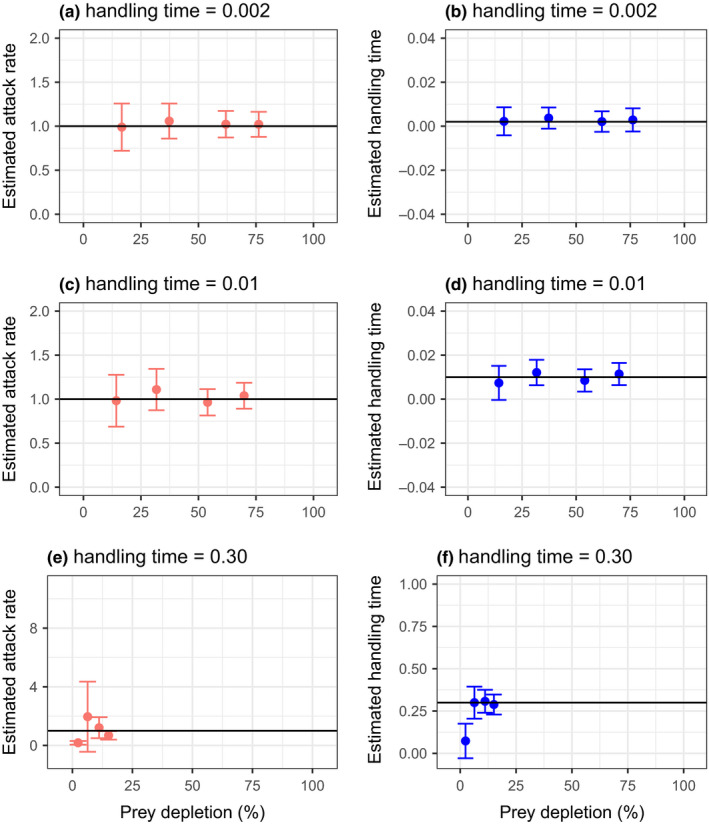
Functional response parameter estimates (attack rates: a, c, e; handling times, b, d, f) modelled from consumption simulations across different prey depletions (total prey eaten divided by total density) and input handling time values. Input attack rates were fixed at 1.00. Estimates are ±*SE* and the solid lines represent the input parameter value. Note differences in axes scaling.

When using the population dynamic model to simulate prey depletion across input parameter values and prey densities mimicking our experimental design, we found that estimated values of *a* and *h* were overall statistically similar to input “known” values of both *a* and *h* (duration: Figures A1 and A2; depletion: Figures A3 and A4). These included scenarios of total depletion at lowest prey densities, as per our experimental data. For instance, prey depletion was total (100%) for the scenario *a* = 5, *h* = 0.01 at prey densities 2, 4, 8, and 16 for experimental durations of 1 hr and more. Estimated attack rates and handling times always overlapped input values, with one exception at a duration shorter than our experiment (0.2 hr: *a* = 1.00, *h* = 0.30) (Figure A2e,f), and where prey depletion was low (Figure A4e,f). Accordingly, increasing experimental duration, and thus prey depletion, did not significantly bias estimates across a broad range of input *a* and *h* values.

## 4 DISCUSSION

This study demonstrates the influence of intraspecific shifts in body size ratios during ontogeny on the strength of predator–prey interactions. Biotic processes such as predation exert profound influence upon the stability and functioning of ecosystems (Brose et al., 2019; Wasserman & Froneman, 2013), and interaction strengths between trophic groups have been shown to scale with relative body size (Barrios‐O'Neill et al., 2016; Brose et al., 2019; Schröder, Kalninkat, & Arlinghaus, 2016). Given that body size ratios between predators and prey are highly variable, examinations of ecological impacts across body size variations are crucial to predict community outcomes (Asquith & Vonesh, 2012; Brose et al., 2019; Jennings & Warr, 2003). However, studies quantitatively examining the influence of body size variations on the strength of predator–prey interactions are lacking at the intraspecific level. As a result, there has been a relatively poor understanding of how consumer–resource participant heterogeneity can modulate predation effects (see Barrios‐O'Neill et al., 2016; Gallagher, Brandl, & Stier, 2016; McCoy et al., 2011; Uiterwaal, Mares, & DeLong, 2017). This, in turn, limits insights into potential tradeoff dynamics between paired predators and prey given often asynchronous ontogenic development (e.g., Olson, 1996). The results here demonstrate that scalings of predator–prey body size can alter impacts from invasive fish toward their prey. Furthermore, they show that body size scalings can interact and manifest differently between predator species and potentially drive differential invader impacts.

We found that all size classes of both bluegill and largemouth bass exhibited a saturating type II functional response toward each size class of tilapia prey. However, there were marked differences in predation rates between the species, and predator–prey interactions differed within intraspecific body size ratios. Predation by largemouth bass was generally greater than in bluegill across all body size ratios examined here which was expected given the reported voracity of bass (e.g., Alexander et al., 2014). However, the higher predation of largemouth bass was most pronounced at small and intermediate predator sizes, and became less important where large individuals of both predator fish species were used. Indeed, there was no clear difference between feeding rates of medium‐ and large‐sized largemouth bass, while raw consumption by bluegill always increased incrementally with body size. Although diets of many fish species change with ontogeny, for instance due to temporal variations in resource supplies or changeable physiological demands (Werner & Gilliam, 1984), the timings of shifts to piscivory can vary considerably between species. Largemouth bass can be piscivorous from as early as age‐0 (Pelham et al., 2001), while shifts in bluegill piscivory may occur much later, owing to littoral zone occupancy during early ontogenic stages (Mittelbach, 1981). Thus, consumptive differences between the species presented here align with their documented feeding ecology, with interspecific differences greatest at small predator sizes. Accordingly, our results help quantitatively inform the nature of dietary shifts in empirical contexts and also help predict the ecological impacts of these invaders across size ratio differences. In particular, the high predatory impacts of largemouth bass corroborate well‐established field impacts, whereby native prey populations are frequently reduced and other fish populations extirpated, while bluegill effects may be less evident (Ellender et al., 2011; Weyl, de Moor, Hill, & Weyl, 2010).

Overall, considering the effects of predator and prey body size separately, functional response magnitudes were generally highest toward smaller prey across all size classes of each predator species. Likewise, functional responses by larger predators were generally higher than in smaller body size classes indicating a higher maximum predation rate for larger predators compared to smaller ones. While smaller prey sizes exhibit reduced reaction distances and escape speeds (Hansen & Wahl, 1981; Rodgers et al., 2014), larger predators frequently demonstrate enhanced capture rates due to improved visual acuity, increased gape, and greater swim speeds (Christensen, 1996; Graeb et al., 2006; Miller et al., 1993). Nevertheless, interactive complexity between relative predator–prey body sizes can emerge due to inefficiency in capturing prey that are relatively large or small (Brose, 2010; McCoy et al., 2011). On the other hand, sustained ecological impacts irrespective of predator size class have been shown in other studies (see Gallagher et al., 2016). Here, differences in the magnitude of functional responses of both predator species, regardless of predator size, were less marked toward large‐sized prey. This may reflect general handling constraints associated with this prey size for the two predator species.

Maximum attack rates of predators are often observed at intermediate predator–prey body size ratios under certain conditions (Barrios‐O'Neill et al., 2016). The present study demonstrates emergent effects of body size between predator–prey pairings on attack rates (i.e., capture/search efficiencies), with attack rates tending to scale unimodally. Small predators exhibited greater attack rates toward small‐ and medium‐sized prey, while large bluegill and intermediate largemouth bass displayed the greatest capture efficiencies toward large prey. In turn, attack rates for both predators were lowest by large individuals toward small prey and by small predators toward large prey. This provides further evidence for the importance of predator–prey body size ratios for interaction strengths, with predators often exhibiting greater capture efficiencies toward similarly scaled prey in respect to size. Moreover, the heightened attack rate of medium‐sized largemouth bass toward large prey may suggest that capture efficiencies instead peak under greater prey sizes than tested in the current study for large individuals of this species. Indeed, largemouth bass attack rates peaked unimodally under lower predator–prey body size ratios than bluegill in the present study. Given that high attack rates are conducive to greater ecological impact potential at low resource densities (Bollache, Dick, Farnsworth, & Montgomery, 2008; Cuthbert, Dickey, Coughlan, Joyce, & Dick, 2019; Dick et al., 2013), low prey density predatory impacts may be reduced toward relatively small or large prey. For large prey in particular, these patterns may elicit greater stability through refugia, given the greater range of capture efficiencies demonstrated toward this size class. Importantly, however, as with many comparative functional response studies (e.g., Sheath et al., 2018; Wasserman, Alexander, Dalu, et al., 2016), the present study employed a nonreplacement experimental design which resulted in consistently high prey depletion under certain treatments. While the statistical measures applied account for prey depletion, feeding interactions were likely constrained at low prey densities. Contrastingly, replacement designs could allow for improved attack rate estimation (i.e., initial curve slope) and better discrimination between experimental treatment groups (Dick et al., 2014). Regardless, nonreplacement experimental designs such as ours provide useful comparative insights into context dependencies affecting trophic interactions. Furthermore, our analyses through simulations indicated that increasing prey depletion through longer experimental duration did not cause bias in parameter estimation using the random predator equation, across a range of starting attack rate and handling time values. Accordingly, prey depletion likely did not impact upon the robustness of our empirical results, even where prey were totally depleted at some densities.

Patterning of handling times was more consistent in our study system than that of attack rates. We found that handling times unsurprisingly increased concurrently with prey size indicating that more time is needed to handle and digest larger prey. Handling times for both predators related negatively to increasing predator–prey body mass ratios. As prey size increased, medium‐ and large‐sized bluegill generally exhibited greater similarity in handling times, while small bluegill displayed significant handling constraints relative to largemouth bass. Conversely, medium and large largemouth bass handling times were less consistently significantly different. These traits may again reflect a relatively early ontogenic shift to piscivory in largemouth bass (Pelham et al., 2001). As with attack rates, a greater range of handling times was displayed toward large‐sized prey, likely driven by size‐related handling constraints. Nonetheless, further research that presents different prey types simultaneously is required to elucidate selectivity or switching processes which may stabilize trophic interactions (e.g., Cuthbert, Dickey, McMorrow, Laverty, & Dick, 2018), given that prey types were provided singularly in the present study.

The application of functional responses offers useful insights into the density dependencies of interaction strengths comparatively (Jeschke, Kopp, & Tollrian, 2002; Wasserman, Alexander, Weyl, et al., 2016). While such comparisons can be difficult to parallel with empirical community dynamics (Vonesh, McCoy, Altwegg, Landi, & Measey, 2017a, 2017b), functional responses offer comparative insights into the influence of context dependencies on predatory impacts (Sentis & Boukal, 2018; Sheath et al., 2018; Wasserman, Alexander, Dalu, et al., 2016). Our findings demonstrate effects of body size ratios on the predatory interaction strengths between piscivorous fishes and their prey. Moreover, we show clear consumptive differences between two key invaders which may relate to differential impacts on prey across their ontogeny. While traits other than body size can influence interaction strengths (e.g., temperature: Cuthbert, Dick, Callaghan, & Dickey, 2018; Englund, Öhlund, Hein, & Diehl, 2011; Sentis, Hemptinne, & Brodeur, 2013), effects of body size have been shown to be particularly pervasive across trophic and taxonomic groups (Barrios‐O'Neill et al., 2016; Brose et al., 2019). The present study furthers these findings, highlighting the importance of both inter‐ and intraspecific differences in predator–prey body size ratios for trophic interactions. Therefore, asynchronous ontogenic development between predators and prey likely affect community outcomes with respect to population stabilities. We thus propose that considerations for the intra‐ and interspecific scaling of body size in consumer–resource systems can greatly enhance our predictive capacity for community interactions, invasive species ecological impacts, and ecosystem structuring.

## CONFLICT OF INTEREST

The authors declare no conflicts of interest.

## AUTHOR CONTRIBUTIONS

**Ross N. Cuthbert:** Formal analysis (lead); validation (lead); visualization (lead); writing – original draft (lead); writing – review & editing (lead). **Ryan J. Wasserman:** Conceptualization (equal); data curation (equal); investigation (equal); methodology (equal); writing – review & editing (equal). **Tatenda Dalu:** Investigation (equal); methodology (equal); writing – review & editing (equal). **Horst Kaiser:** Methodology (equal); resources (equal); writing – review & editing (equal). **Olaf L. F. Weyl:** Conceptualization (equal); funding acquisition (equal); methodology (equal); project administration (equal); resources (equal); supervision (equal); writing – review & editing (equal). **Jaimie T. A. Dick:** Conceptualization (equal); project administration (equal); supervision (equal); writing – review & editing (equal). **Arnaud Sentis:** Formal analysis (equal); methodology (equal); software (equal); visualization (equal); writing – review & editing (equal). **Michael W. McCoy:** Conceptualization (equal); methodology (equal); writing – review & editing (equal). **Mhairi E. Alexander:** Conceptualization (equal); data curation (equal); funding acquisition (equal); investigation (equal); methodology (equal); writing – review & editing (equal). 

## Data Availability

All data from this manuscript are freely available at the Dryad Digital Repository: https://doi.org/10.5061/dryad.7m0cfxppt (Cuthbert et al., 2020).
