# Supplementary figures and images for: Influence of intra‐ and interspecific variation in predator–prey body size ratios on trophic interaction strengths
Source: Ecol Evol. 2020 Jun 1;10(12):5946–62. doi: 10.1002/ece3.6332 (PMC7319243; doi:10.1002/ece3.6332)

(a) attack rate = 0.45

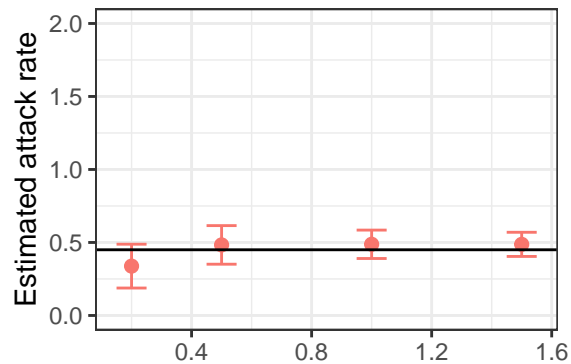

(b) attack rate = 0.45

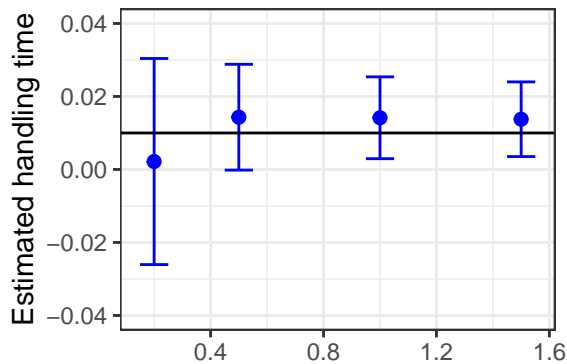

(c) attack rate = 1.00

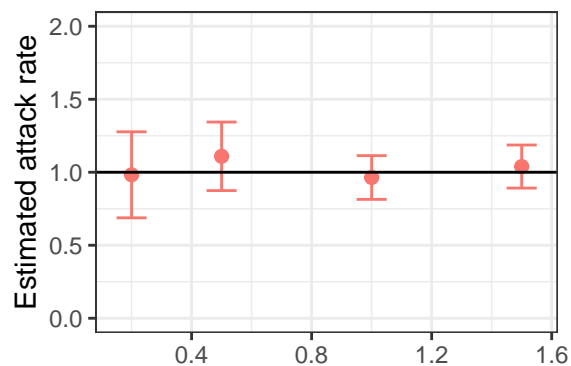

(d) attack rate = 1.00

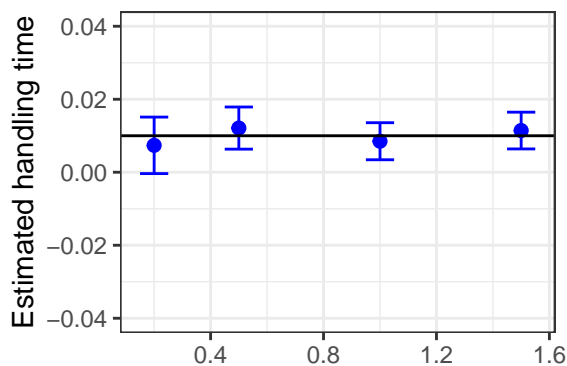

(e) attack rate = 5.00

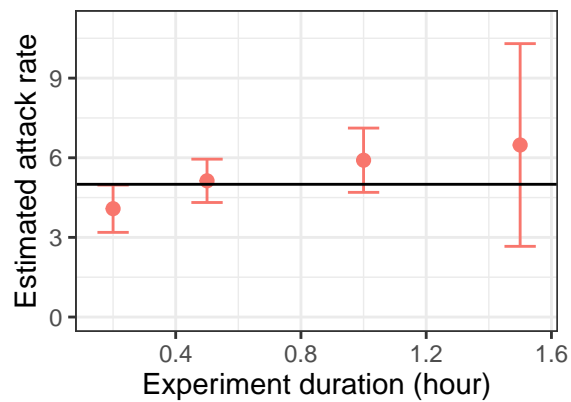

(f) attack rate = 5.00

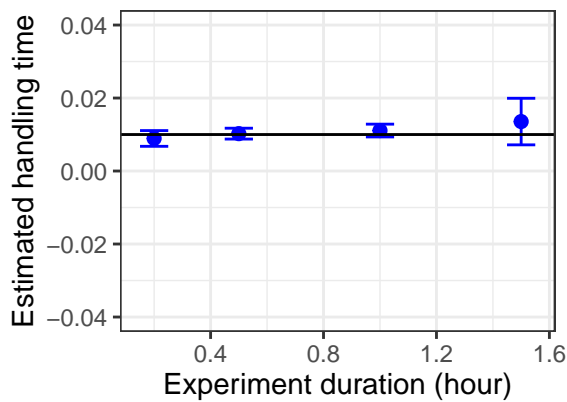

Supplement: Supplementary file 1 [file ECE3-10-5946-s001.pdf]

(a) handling time = 0.002

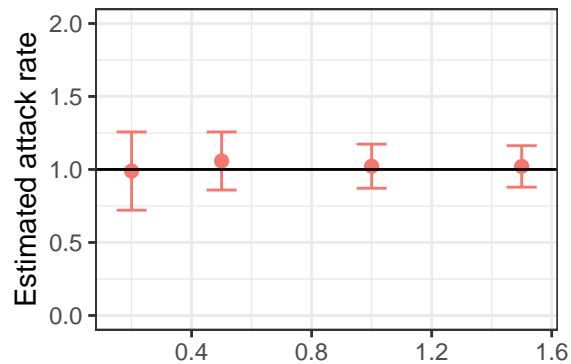

(b) handling time = 0.002

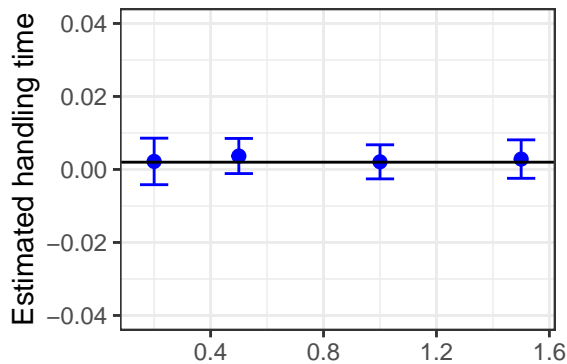

(c) handling time = 0.01

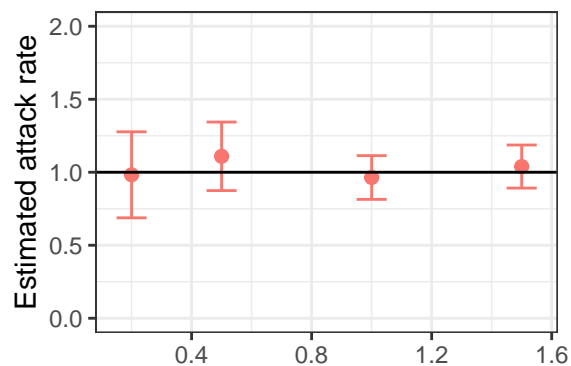

(d) handling time = 0.01

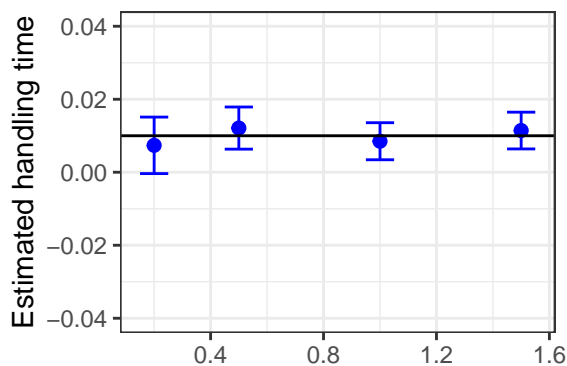

(e) handling time = 0.30

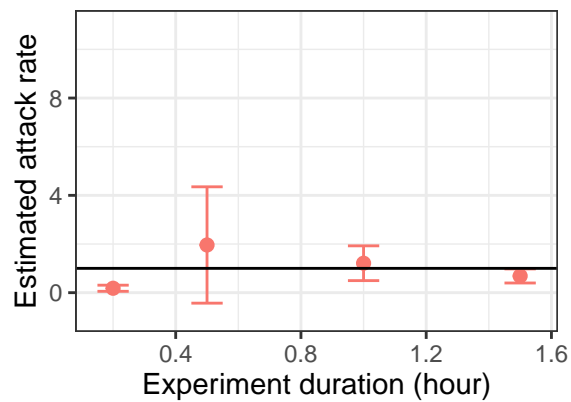

(f) handling time = 0.30

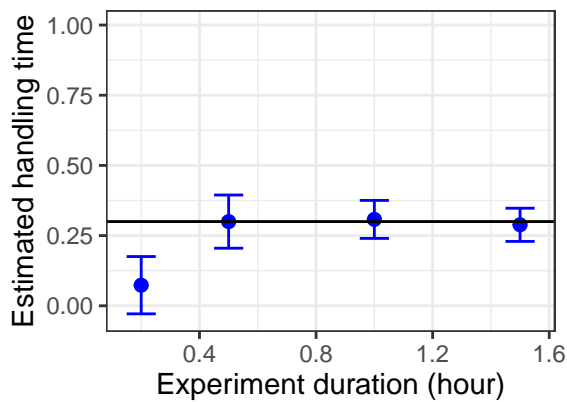

Supplement: Supplementary file 2 [file ECE3-10-5946-s002.pdf]

(a) attack rate = 0.45

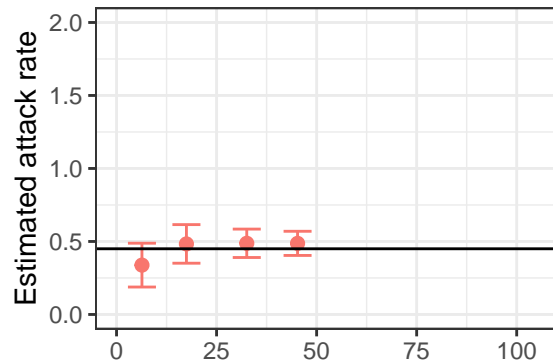

(b) attack rate = 0.45

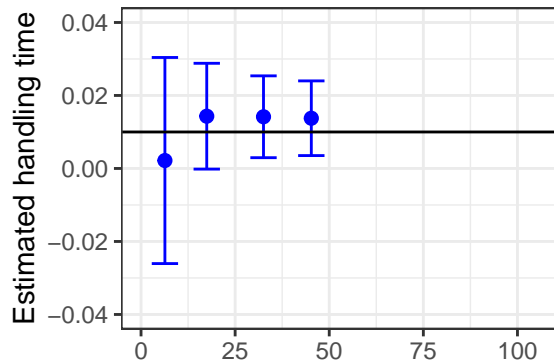

(c) attack rate = 1.00

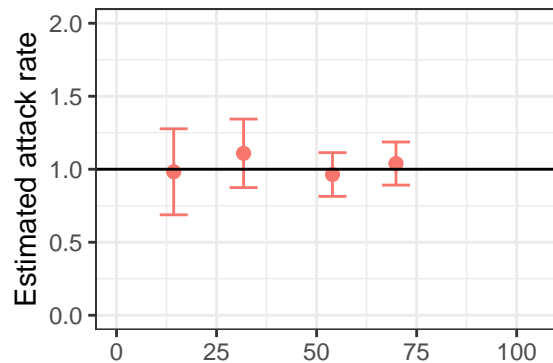

(b) attack rate = 1.00

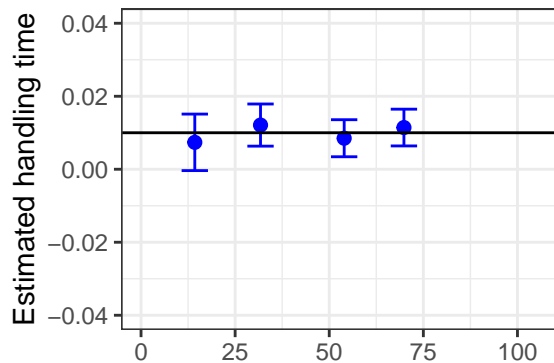

(e) attack rate = 5.00

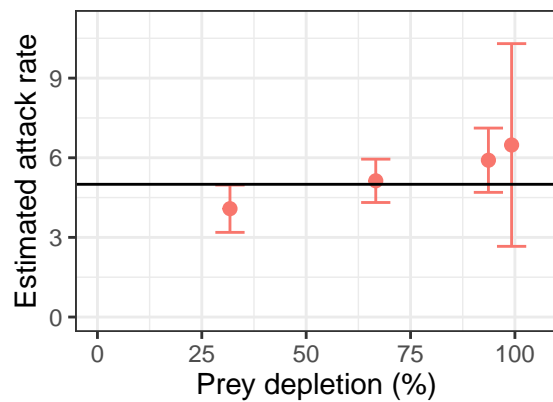

(f) attack rate = 5.00

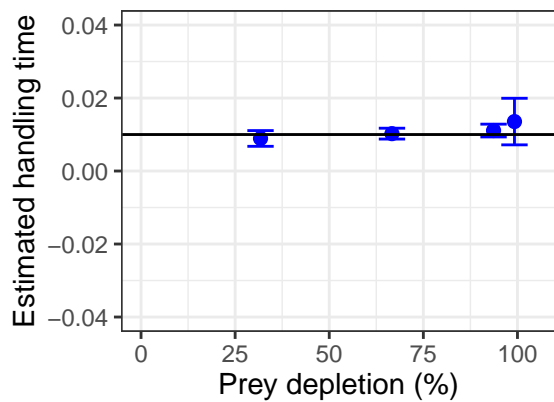

Supplement: Supplementary file 3 [file ECE3-10-5946-s003.pdf]

(a) handling time = 0.002

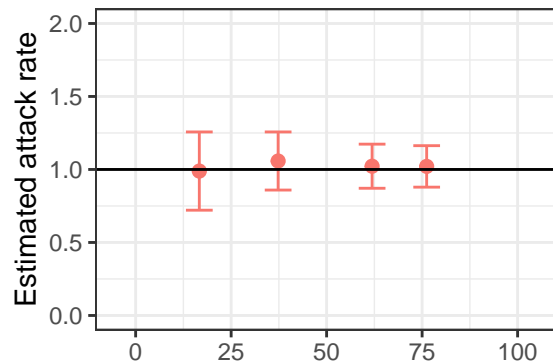

(b) handling time = 0.002

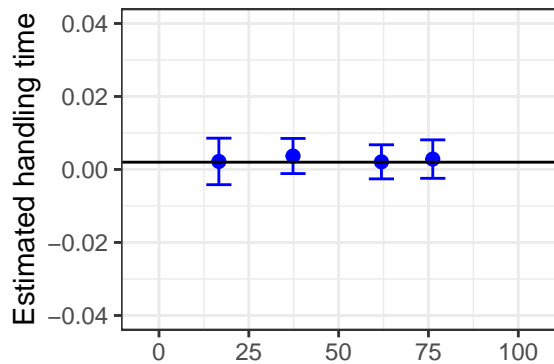

(c) handling time = 0.01

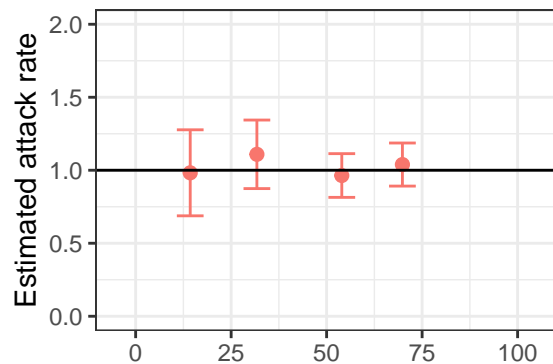

(d) handling time = 0.01

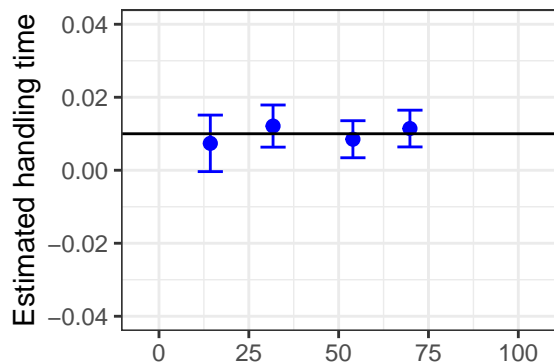

(e) handling time = 0.30

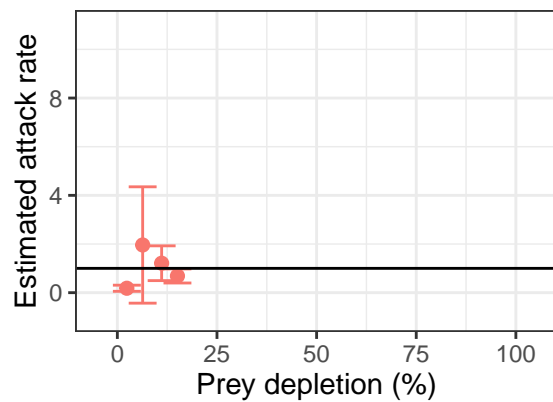

(f) handling time = 0.30

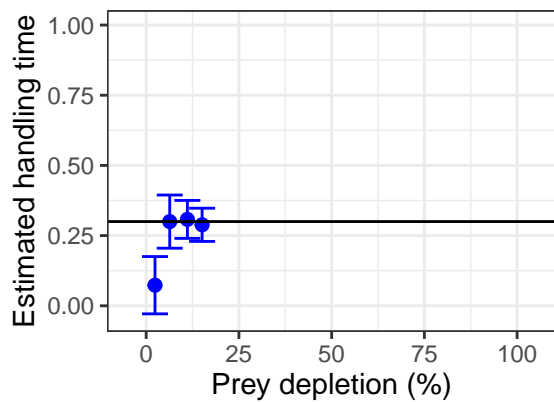

Supplement: Supplementary file 4 [file ECE3-10-5946-s004.pdf]
